# High Atmospheric CO_2_ Concentration Mitigates Drought Effects on *Acanthostyles buniifolius* an Important Grassland Weed in South America

**DOI:** 10.3390/plants11172270

**Published:** 2022-08-31

**Authors:** Tamara Heck, Marcus Vinícius Fipke, Rubens Antonio Polito, Gustavo Maia Souza, Dirceu Agostinetto, Anderson Luis Nunes, Luis Antonio de Avila

**Affiliations:** 1 Department of Crop Protection, Federal University of Pelotas, Campus Universitário, S/N, Capão do Leão 96160-000, RS, Brazil; 2 Department of Botany, Federal University of Pelotas, Campus Universitário, S/N, Capão do Leão 96160-000, RS, Brazil; 3 Department of Crop Protection, Federal Institute of Rio Grande do Sul, Rodovia RS 135, Km 32,5 Distrito Eng. Luiz Englert, Sertão 99170-000, RS, Brazil

**Keywords:** climate change, drought stress, Pampa Biome, Chirca

## Abstract

The differential growth and yield response of plant species to rising carbon dioxide concentrations and climatic change may alter species diversity within biomes. The Pampa Biome in South America is an important grassland biome of agronomic and environmental importance. *Acanthostyles buniifolius* (Chirca) is one of the most important weeds in natural pasture areas widely distributed in southern South America and can adversely affect livestock production. The current study was designed to identify possible responses of Chirca to CO_2_ concentration ([CO_2_]) and drought that would indicate higher adaptation and potential proliferation within the Pampa Biome. Chirca plants were cultivated at two CO_2_ concentrations (400 (*a*[CO_2_]) and 700 (*e*[CO2]) µmol mol^−1^) and two water conditions (under water restriction—15% of the pot capacity; and plants without water restriction—pot capacity). Besides growth parameters, we also determined water potential (ѱw), relative water contents (RWC), proline, glycine betaine, total soluble sugars, hydrogen peroxide, lipid peroxidation, superoxide dismutase (SOD), ascorbate peroxidase (APX) activity, chlorophyll A and B, carotenoids and root dry mass (RDM). Plants exposed to *e*[CO_2_] are more efficient in water use and have a greater increase in root dry mass, enabling greater adaptation to climate-induced droughts. Among the biochemical changes observed in the plants under drought stress, the accumulation of proline, glycine betaine, and total soluble sugars were the most evident mechanisms allowing plants to tolerate drought stress by osmotic adjustment.

## 1. Introduction

The Pampa Biome is an important and extensive (~800,000 km^2^) grassland ecosystem in South America, which covers areas in southern Brazil, Paraguay, Argentina, and Uruguay [1]. Historically, the Pampa Biome has been used for livestock production, especially cattle, sheep, goats, and horses [2,3], presenting a high degree of biodiversity [4,5]. Therein, Asteraceae represents the family with the greatest species richness and can form dense populations, with several shrubs or sub-shrubs species, such as *Acanthostyles buniifolius* (Hook. and Arn.) (common name Chirca); however, livestock rarely consumes these plants [2,6]. The use of extensive livestock in native pastures represents great economic importance in Brazil. However, increased weed infestation has contributed to the degradation of native grasslands and a decrease in quality and animal support capacity [7].

Chirca, among the most troublesome weeds in the native pasture in the Pampa Biome [8,9], belongs to the Asteraceae family and the Milleriaceae tribe [10]; it is a perennial, shrub-sized, highly branched plant 0.8–2.0 m in height and only reproduces sexually by seeds. It blooms in March and April, and the fruits are achenes, which, due to their hairy pappus, provide wide dispersion, both by wind and animals [11,12]. It is well adapted to the poor and acidic soils of the region [11,12]. Due to these characteristics, its management can be difficult [4].

Recent and projected increases in atmospheric CO_2_, and the consequent changes in global climate, including temperature and rainfall, are likely to change fundamental aspects of plant biodiversity within a community, depending on the response of individual plant species [13,14]. Understanding differential plant responses to increasing CO_2_ and changes in drought or temperature are key aspects in determining changes in plant communities and their long-term functionality [15].

Climate change is also the cause of change in rainfall patterns, which also reflects many effects on plants [16]. Droughts can also occur in natural conditions, but environmental changes have vastly accelerated hydrological processes to make them faster and more severe [17]. These changes are progressive and projected to intensify for the end of the century [18]. Drought stress is a limiting and critical factor for plant survival, affecting many physiological and biochemical processes [19]. Thus, plants have evolved mechanisms to tolerate water stress ranging from the translation of stress signals to gene regulation, and from metabolic effects to whole plant morpho-physiological changes [20].

Physiological changes under water deficit conditions, such as stomatal closure, reducing water loss by transpiration, and osmotic adjustments, are in the front of the plant responses to drought [21]. However, as a side effect, when stomatal conductance is reduced, the internal leaf concentration of CO_2_ is reduced as well, resulting in a drop in photosynthetic CO_2_ assimilation and, consequently, reducing the availability of energy from photochemical activity, which increases the production of reactive oxygen species (ROS) [22]. Thus, prolonged periods of drought can lead to the accumulation of ROS and, therefore, induce oxidative stress by lipid peroxidation of the plasma membrane, as well as oxidation of the entire metabolic apparatus [23,24].

On the other hand, plants have an effective antioxidant defense system, which encompasses the action of antioxidant enzymes such as superoxide dismutase (SOD), ascorbate peroxidase (APX), catalases (CAT), and guaiacol peroxidases, as well as non-enzymatic systems, which prevent or reduce the damage caused by oxidative stress [25] as the accumulation of proline and total soluble sugars in a more advanced stage of water deficit [20]. Proline acts as an osmoprotector that stabilizes membranes and maintains the conformation of cytosolic enzymes to survive water stress [26]. Likewise, the accumulation of sugars and amino acids is related to the mechanism that allows plants to increase their tolerance to low water availability by allowing plants to maintain cellular turgor pressure [27].

Given the troublesome nature of Chirca in the Pampa Biome, the effect of rising [CO_2_] and drought stress is worth a closer examination. Such examinations are fundamental in determining the adaptive capacity of this species and its potential expansion within the Pampa. Therefore, the objective of this study was to assess the role of projected CO_2_ increases on the vegetative and reproductive response of Chirca to drought and identify the possible mechanisms that lead Chirca to adapt to an increase in CO_2_ and drought stress.

## 2. Material and Methods

### 2.1. Plant Material and Growth Conditions

Seeds of Chirca were collected in Capão do Leão—RS, in a pasture area. Chirca seeds were sown in plastic boxes and plant growth chambers in a controlled environment at 28/25 °C (day/night) and 12 h photoperiod (day/night). Plants emerged seven days after sowing and were transplanted two weeks later from plots. The experimental units consisted of one plant and 8 L pots, with a volume of 0.042 m^2^ of sandy loam soil, placed inside the “open-top chamber” (OTCs). The experiment was conducted in growth chambers with automatic control of atmospheric CO_2_ concentration OTCs. Factor A consisted of two levels of CO_2_, ambient (*a*[CO_2_]) at 400 ± 50 μmol mol^−1^ and elevated (*e*[CO_2_]) at 700 ± 50 μmol mol^−1^. Factor B represented water restriction conditions and the control (without drought). The water deficit consisted of irrigation suppression until the plants reached 15% of their pot capacity (Cw). Cw was determined according to Dos Santos et al. (2015), with some methodological adaptations. The Cw was calculated using the fresh mass of the soil after water saturation (Cfm) and the dry mass (Cdm) after drying the soil until it reached the constant weight and applied in the equation Cw = (Cfm − Cdm)/Cfm × 100.

**Figure 1:**
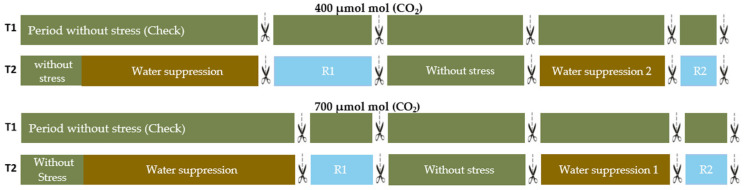
Distribution of treatments, where T1 = fully irrigated T2 = experienced water deficit, Water 1 suppression = first water suspension in the growing season, Water suppression 2 = second water suspension when plants have reached 50% of the reproductive period. R1 and R2 = rehydration period.

The plants were kept at pot capacity (full irrigation) until the beginning of the water deficit treatment imposed at 120 days after emergence and a second deficit imposed at 246 days after emergence, comprising the vegetative and reproductive stages, respectively (Figure 1). The beginning of the reproductive stage was considered when 50% of the plants started the flowering period. Firstly, plants were stressed by water deficit until they reached 15% of pot capacity, where for Chirca it corresponded to 15 and 10 days for *e*[CO_2_] and *a*[CO_2_], respectively. After the first stress, the plants underwent a recovery period until 246 days after emergence. For the second water stress, the plants were kept under water deficit until again reached 15% of pot capacity, a condition that lasted 7 and 6 days for *e*[CO_2_] and *a*[CO_2_], respectively.

During the period of water deficit, the pots were monitored by daily weighing, at consistent daily times, and an additional reading was performed with the soil moisture sensor (TDR—Time Domain Reflectometer–Trace System). For the control treatment, water was replaced, assuming the maintenance of pot capacity at 15% and 100%, considering 1 mL = 1 g. After reaching 15% Cw, the plants were collected, and for the treatment of water stress, the total suspension of water was carried out. Two sampling times were carried out, on the day of water deficit (15% Cw) and rehydration (R1 = 4 days after the resumption of full irrigation; R2 = two days after the resumption of irrigation) and compared to the fully irrigated control. After collection, the samples were stored in an ultra-freezer at −80 °C for analysis.

The following parameters were measured following water stress and rehydration: water potential (ѱw), relative water contents (RWC), proline, glycine betaine, and total soluble sugars, hydrogen peroxide, lipid peroxidation, superoxide dismutase (SOD), ascorbate peroxidase (APX) activity, chlorophyll A and B, and carotenoids, such as root dry mass. A summary of the results of the variables analyzed at both stages of development are provided in the Supplementary Material (Tables S1 and S2). The design was completely randomized in a bi-factorial scheme with four replications (Figure 1).

### 2.2. Biochemical Parameters

Determination of hydrogen peroxide and lipid peroxidation: leaf tissues (±0.25 g) were macerated with liquid nitrogen and homogenized with 0.1% trichloroacetic acid (TCA) (*w*:*v*). The homogeneous mixture was centrifuged (12,000× *g*, 4 °C, 20 µm), and the supernatant was used to determine the H_2_O_2_ content according to ref. [28]. Lipid peroxidation was determined as described by ref. [29], using thiobarbituric acid (TBA), determining malondialdehyde (MDA) as the final product of lipid peroxidation. The MDA–TBA complex was calculated from the extinction coefficient (ε = 155 × 103 M^−1^ cm^−1^).

Photosynthetic pigments: leaves (±0.02 g) were used. The samples were soaked in 7 mL of dimethylsulfoxide (DMSO) solution neutralized with 5% calcium carbonate, as described by ref. [30] with some methodological adaptations. Then, the test tubes were heated in a water bath at 65 °C for 4 h. After reaching room temperature, the absorbance of the homogenate was determined at the wavelengths of 665, 649, and 480 nm to chlorophyll A, chlorophyll B, and carotenoids, respectively (Lichtenthaler e Buschmann 2001).

Super oxide dismutase activity was determined in leaves (±0.25 g) as described by ref. [31], which were ground in a mortar and pestle with liquid nitrogen, containing 5% (*w*:*v*) of PVPP and buffer of 100 mM potassium phosphate, pH 7.8, containing ethylenediaminetetraacetic acid (EDTA) and 20 mM sodium ascorbate. The homogeneous mixture was centrifuged at 12,000× *g* (for 20 min at 4 °C). An aliquot of the supernatant was used as a crude enzyme extract. The activity of SOD was determined according to the inhibition of nitro-blue-tetrazolium (NBT) staining at 560 nm. The oxidation activity of ascorbate peroxidase (APX; EC 1.11.1.11) was determined by ascorbate oxidation at 290 nm.

Proline and total soluble sugar content were determined in leaf plant tissues (±0.5 g) using leaves at stage of growth. Leaves were ground in a mortar with 2 mL of MCW (methanol, chloroform, and water in a ratio of 12:5:3), as described by ref. [32]. Proline dosage was determined by the ninhydrin method, according to ref. [33] with some methodological adaptations. This biphasic suspension, obtained following this method, contains 1 mL of the upper phase and was collected and analyzed in a spectrophotometer at 520 nm. The absorbance obtained was compared with the standard curve for proline, and the results were expressed in µmol g^−1^ of fresh weight. The total soluble sugars were determined by the anthrone method [34].

Glycine betaine content: plant tissues at certain stages of growth were ground in a mortar (±0.25 g), and 10 mL deionized water was added, leaving the extract under stirring (230 rpm) for 24 h at 25 °C according to ref. [35] with some methodological adaptations. The samples were filtered, an aliquot of 0.5 mL of the extract was added at a 1:1 ratio of sulfuric acid (H_2_SO_4_) 2N, homogenized, and an aliquot of 0.5 mL was removed in polypropylene microtubes and kept on ice for 1 h. It was added to this aliquot 0.2 mL of potassium iodide (KI-I_2_), vortexed, and stored at 0–7 °C for 16 h. After being thawed and homogenized in a vortex, the sample was centrifuged at 10,000 rpm for 15 min at 4 °C. The supernatant was collected so that the precipitated betaine periodate crystals remained intact for the washing process with 3 mL 1,2-dichloroethane. After 2 h with the crystals dissolved, a 2 mL aliquot was used to read a spectrophotometer at 365 nm.

### 2.3. Estimation of Water Potential and Relative-Water Contents (RWC)

The water potential (Ψw) was determined on the fully expanded leaf of the middle part of the plant. The Ψw was analyzed at predawn and midday, using the Scholander Pressure Pump Chamber^TM^ (SEC-3115-P40G4V, Soil moisture, Santa Barbara, CA, USA). The RWC was determined as described by ref. [36] following the equation RCW (%) = [(fresh mass − dry mass)/(water-saturated mass − dry mass)] × 100.

### 2.4. Biometric Parameters

Root dry mass (RDM): was obtained by weighing the biomass after drying at 65 °C until a constant weight was reached.

### 2.5. Statistical Analysis

Statistical analyses were performed using R software. Before the analysis of variance, the data were tested for normality. When ANOVA identified significant differences, Tukey’s test was applied using 95% confidence intervals.

## 3. Results

### 3.1. Vegetative Stage

#### 3.1.1. Water Relations

**Figure 2:**
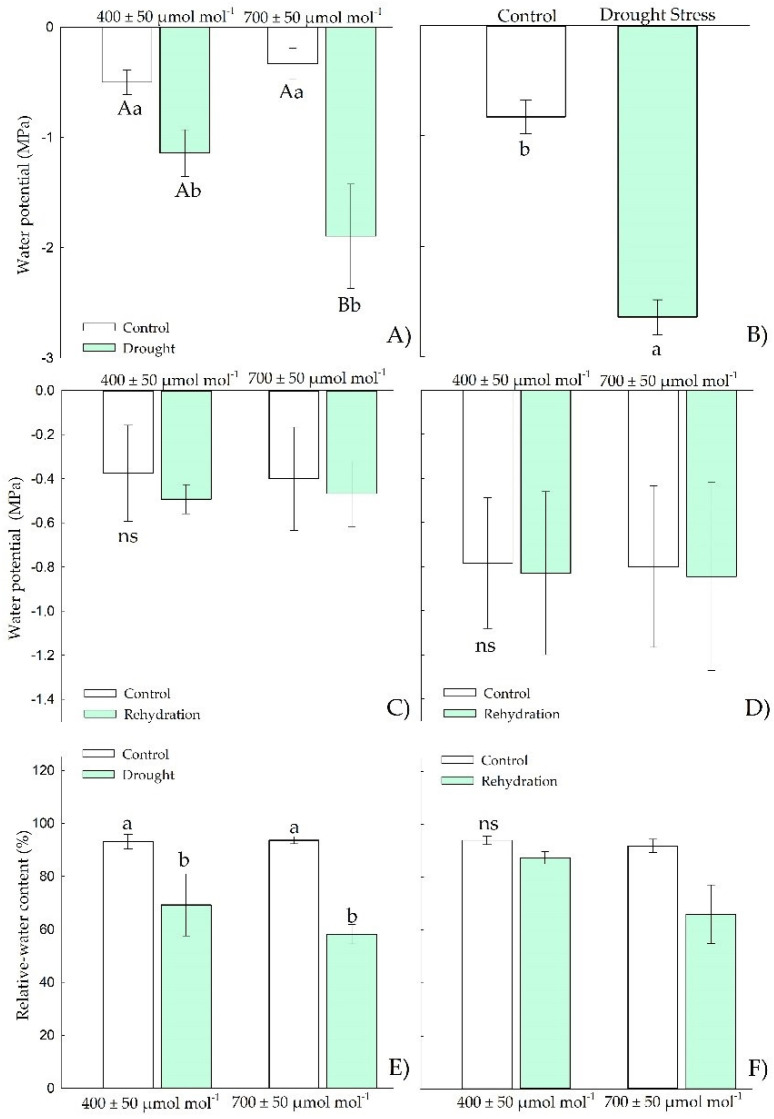
Effects of CO_2_ concentration (*a*[CO_2_] = 400 μmol mol^−1^ and *e*[CO_2_] = 700 μmol mol^−1^) and drought stress in *A. buniifolius* leaf water potential predawn (**A**,**C**), water potential midday (**B**,**D**), relative water content (**E**,**F**). In plants sampled after drought stress (**A**,**B**,**E**), and rehydration (**C**,**D**,**F**) period. Different uppercase letters indicate a significant difference between values in different CO_2_ within the same water treatment, while different lowercase letters indicate significant differences between values of different water treatments within each CO_2_ level by Tukey’s test (*p* ≤ 0.05). ^ns^ denotes non-significance (*p* > 0.05). Error bars represent a 95% confidence interval.

In the vegetative stage, Chirca under drought stress showed reductions in water potential (Figure 2A,B), both in predawn when exposed to *e*[CO_2_] and at midday under drought stress. However, only in the predawn (Figure 2A) the plants showed differences between CO_2_ levels, with more negative water potentials values under *e*[CO_2_]. After the rehydration period (Figure 2C,D), all water potential values were restored to the values of the control plants under constant irrigation.

The relative water content (Figure 2E) was also significantly reduced in plants under drought stress but with no effect of atmospheric CO_2_ concentration. After the rehydration period (Figure 2F), all plants showed values as those of the control plants. The relative water content (Figure 2E) was also reduced in plants under drought stress but with no effect of atmospheric CO_2_ concentration. After the rehydration period (Figure 2F), all treated plants showed values as those of the control.

#### 3.1.2. Osmotic Adjustment Components

**Figure 3:**
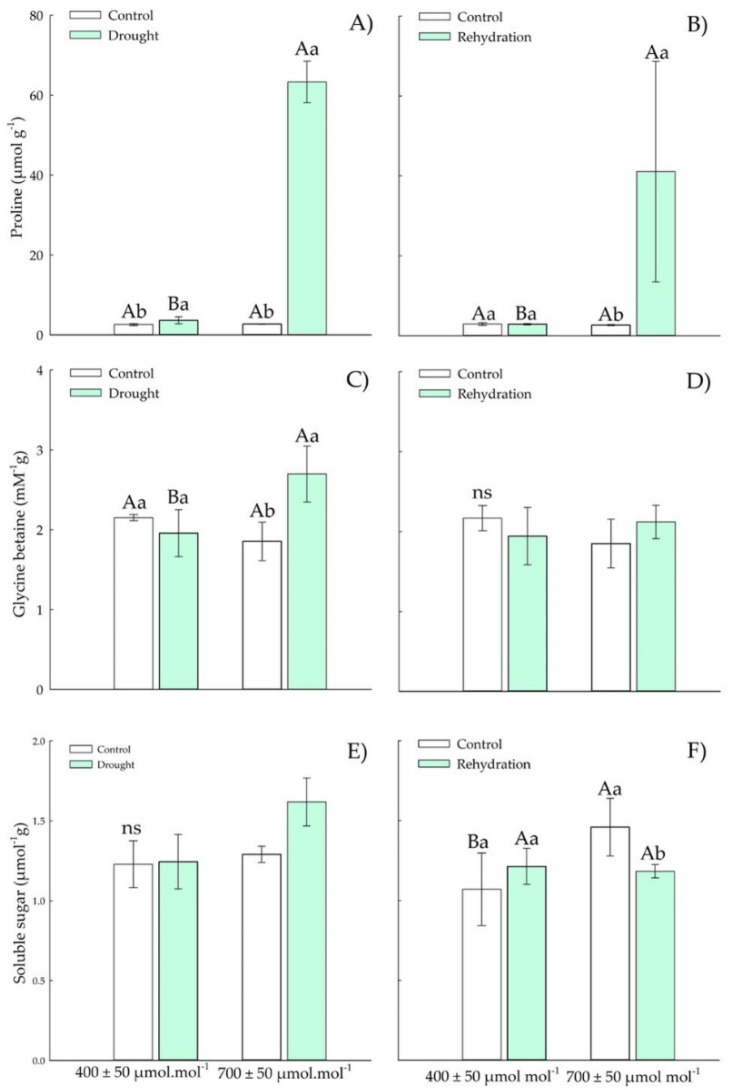
Effects of CO_2_ concentration (*a*[CO_2_] = 400 μmol mol^−1^ and *e*[CO_2_] = 700 μmol mol^−1^) and drought stress in *A. buniifolius* leaf proline (**A**,**B**), glycine betaine (**C**,**D**), soluble sugar (**E**,**F**) content. In plant sampled after drought stress (**A**,**C**,**E**) and rehydration (**B**,**D**,**F**) period. Different uppercase letters indicate a significant difference between values in different CO_2_ within the same water treatment, while different lowercase letters indicate significant differences between values of different water treatments within each CO_2_ level by Tukey’s test (*p* ≤ 0.05). ^ns^ denotes non-significance (*p* > 0.05). Error bars represent a 95% confidence interval.

Chirca plants under drought stress and *e*[CO_2_] showed increases in proline and glycine betaine (Figure 3A,C). The proline content (Figure 3A) in Chirca increased 60-fold in response to drought stress in *e*[CO_2_] compared with control *a*[CO_2_] plants. However, after the rehydration period, only the content of proline (Figure 3B) and total soluble sugars (Figure 3F) showed differences between the CO_2_ levels. The glycine content (Figure 3D) after the rehydration period was restored to the values of the control plants.

The concentration of total soluble sugars (Figure 3F) was also increased in control plants at *e*[CO_2_] after the rehydration period. However, when exposed to drought stress, the concentration of total soluble sugars (Figure 3E), all plants showed values as those of control plants.

#### 3.1.3. Biochemical Parameters—Antioxidants

**Figure 4:**
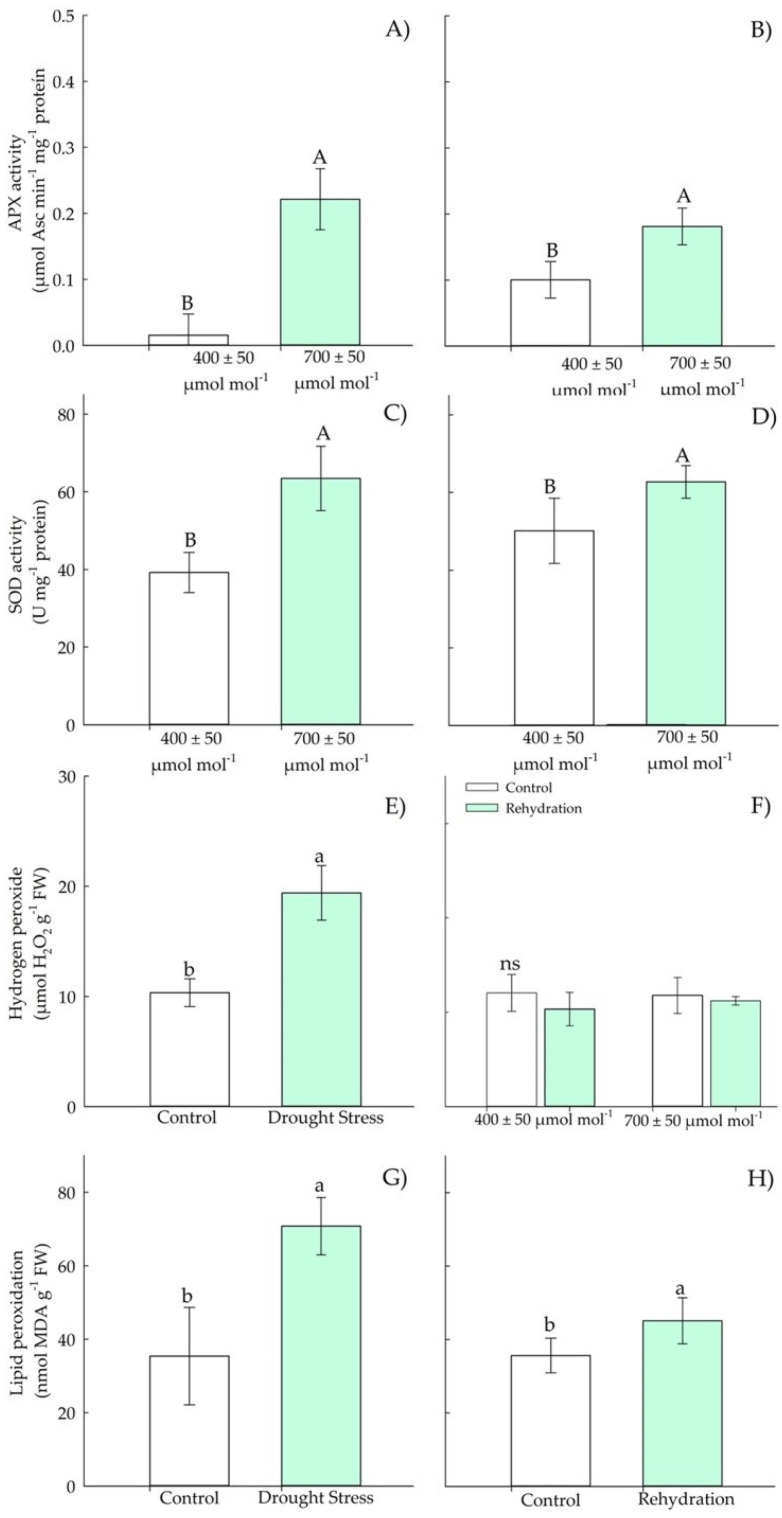
Effects of CO_2_ concentration (*a*[CO_2_] = 400 μmol mol^−1^ and *e*[CO_2_] = 700 μmol mol^−1^) and drought stress in *A. buniifolius* leaf APX activity (**A**,**B**), SOD activity (**C**,**D**), hydrogen peroxide (**E**,**F**), lipid peroxidation (**G**,**H**). In plants sampled after drought stress (**A**,**C**,**E**,**G**) and rehydration (**B**,**D**,**F**,**H**) period. Different uppercase letters indicate a significant difference between values in different CO_2_ within the same water treatment, while different lowercase letters indicate significant differences between values of different water treatments within each CO_2_ level by Tukey’s test (*p* ≤ 0.05). ^ns^ denotes non-significance (*p* > 0.05). Error bars represent a 95% confidence interval.

**Figure 5:**
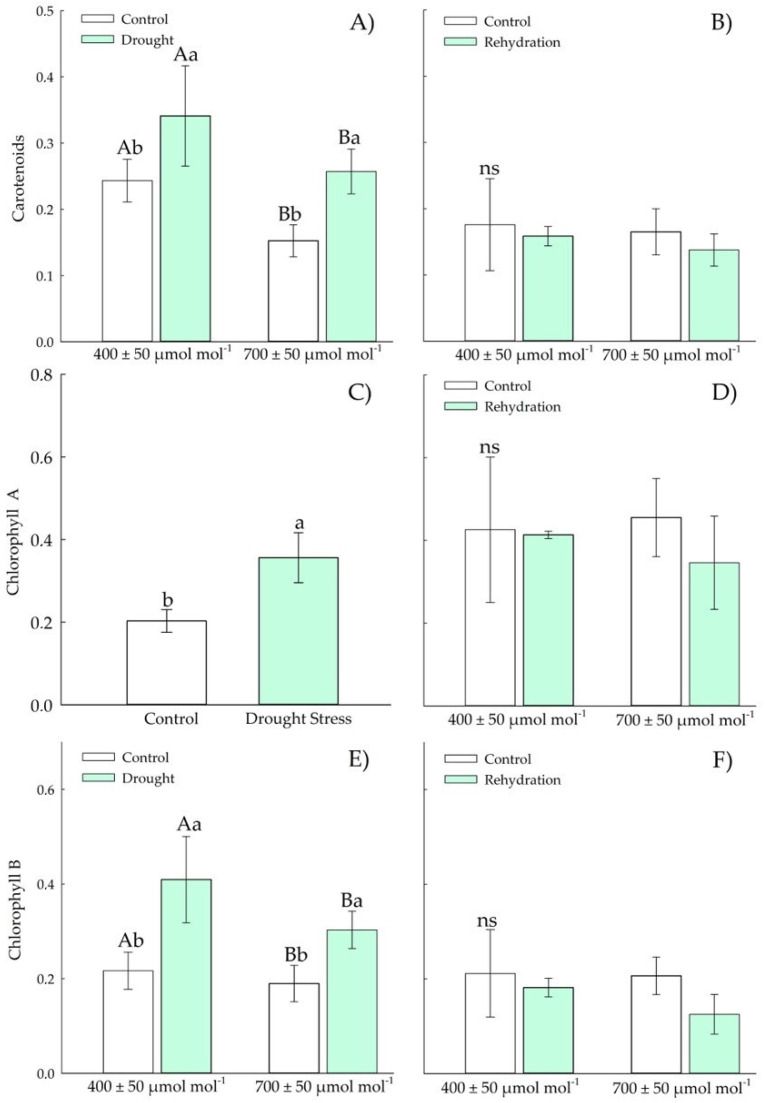
Effects of CO_2_ concentration (*a*[CO_2_] = 400 μmol mol^−1^ and *e*[CO_2_] = 700 μmol mol^−1^) and drought stress in *A. buniifolius* leaf carotenoids (**A**,**B**), chlorophyll A (**C**,**D**), chlorophyll B (**E**,**F**). In plants sampled after drought stress (**A**,**C**,**E**) and rehydration (**B**,**D**,**F**) period. Different uppercase letters indicate a significant difference between values in different CO_2_ within the same water treatment, while different lowercase letters indicate significant differences between values of different water treatments within each CO_2_ level by Tukey’s test (*p* ≤ 0.05). ^ns^ denotes non-significance (*p* > 0.05). Error bars represent a 95% confidence interval.

In the vegetative stage, there was an increase in APX and SOD activity (Figure 4A–D) in Chirca plants subjected to *e*[CO_2_], both in the period of drought stress and in rehydration. The concentration of hydrogen peroxide (Figure 4E), lipid peroxidation (Figure 4G), and chlorophyll A (Figure 5C) increased in plants under drought stress but showed no difference between the concentrations of CO_2_.

The carotenoids (Figure 5A) and chlorophyll B (Figure 5E) concentrations were higher in Chirca plants under *a*[CO_2_] under drought stress. After the rehydration period, carotenoids (Figure 5B), chlorophyll A and B (Figure 5D,F) concentration in all plants showed values as those of control plants.

#### 3.1.4. Root Dry Weight

**Figure 6:**
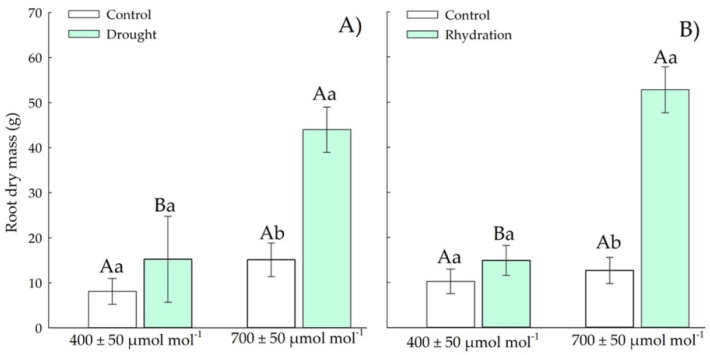
Effects of CO_2_ concentration (*a*[CO_2_] = 400 μmol mol^−1^ and *e*[CO_2_] = 700 μmol mol^−1^) and drought stress in *A. buniifolius* in root dry mass. In plants sampled after drought stress (**A**) and rehydration (**B**) period. Different uppercase letters indicate a significant difference between values in different CO_2_ within the same water treatment, while different lowercase letters indicate significant differences between values of different water treatments within each CO_2_ level by Tukey’s test (*p* ≤ 0.05). ^ns^ denotes non-significance (*p* > 0.05). Error bars represent a 95% confidence interval.

Chirca in the vegetative stage under drought stress and *e*[CO_2_] showed 2.8-fold more RDM (Figure 6A) than the other treatments. After the rehydration period, plants in *e*[CO_2_] showed 3.7-fold more root growth (Figure 6B) than the other treatments.

### 3.2. Reproductive Stage

#### 3.2.1. Water Relations

**Figure 7:**
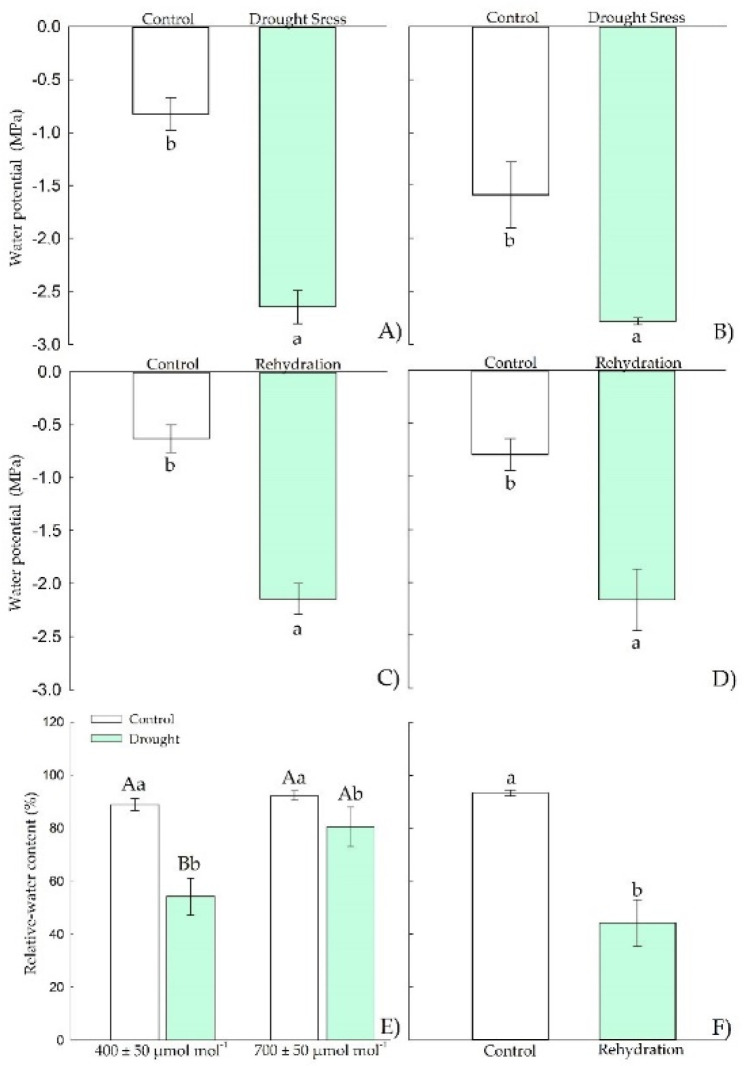
Effects of CO_2_ concentration (*a*[CO_2_] = 400 μmol mol^−1^ and *e*[CO_2_] = 700 μmol mol^−1^) and drought stress in *A. buniifolius* leaf in water potential predawn (**A**,**C**); water potential after midday (**B**,**D**), relative water content (**E**,**F**). In plants sampled after drought stress (**A**,**B**,**E**) and rehydration (**C**,**D**,**F**) period. Different uppercase letters indicate a significant difference between values in different CO_2_ within the same water treatment, while different lowercase letters indicate significant differences between values of different water treatments within each CO_2_ level by Tukey’s test (*p* ≤ 0.05). ^ns^ denotes non-significance (*p* > 0.05). Error bars represent a 95% confidence interval.

At the early reproductive stage, Chirca plants under drought stress showed reductions in water potential values, both in predawn and midday (Figure 7A,B), but without any effect of atmospheric CO_2_ concentration. After rehydration, the water potential values significantly reduced at predawn and midday, but with no CO_2_ effect.

The relative water content was also reduced in plants under drought stress (Figure 7E), independent of CO_2_ levels. After the rehydration period, the relative water content (Figure 7F) was reduced in plants subjected to rehydration, but without any effect of atmospheric CO_2_ concentration.

#### 3.2.2. Osmotic Adjustment Components

**Figure 8:**
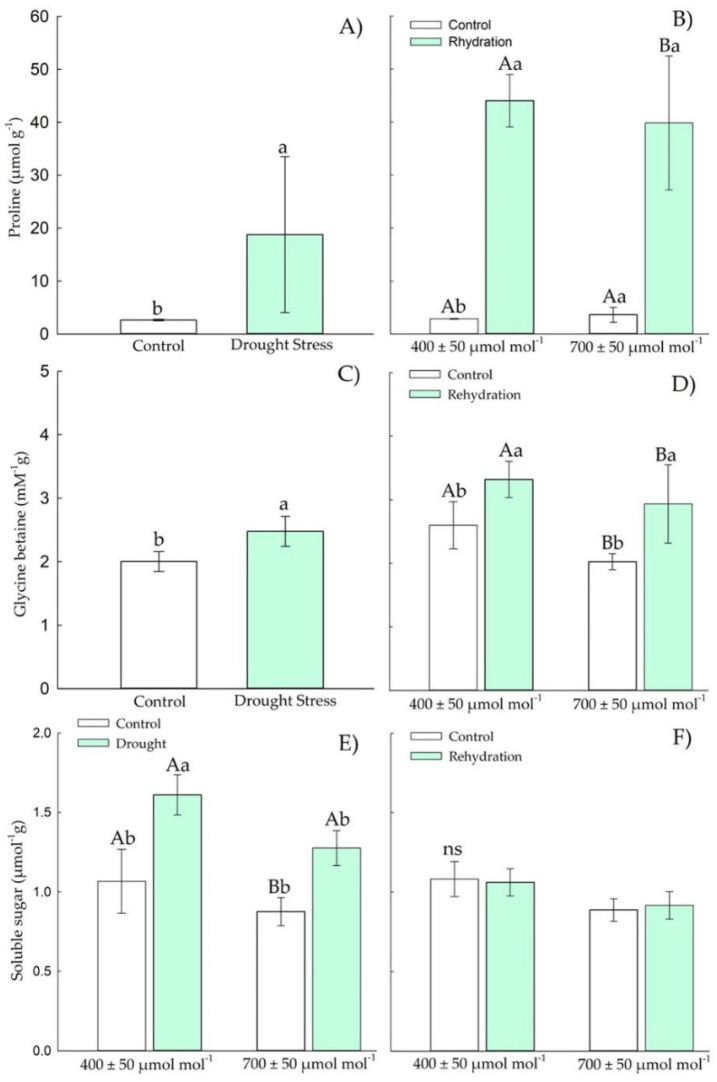
Effects of CO_2_ concentration (*a*[CO_2_] = 400 μmol mol^−1^ and *e*[CO_2_] = 700 μmol mol^−1^) and drought stress in *A. buniifolius* leaf proline (**A**,**B**), glycine betaine (**C**,**D**), soluble sugar (**E**,**F**). In plants sampled after drought stress (**A**,**C**,**E**) and rehydration (**B**,**D**,**F**) period. Different uppercase letters indicate a significant difference between values in different [CO_2_] within the same water treatment, while different lowercase letters indicate significant differences between values of different water treatments within each [CO_2_] level by Tukey’s test (*p* ≤ 0.05). ^ns^ denotes non-significance (*p* > 0.05). Error bars represent a 95% confidence interval.

In the reproductive stage, Chirca plants under drought stress showed increases in proline and glycine betaine (Figure 8A,C), but with no effect of CO_2_ concentration. After rehydration, the proline content (Figure 8A) in Chirca increased by 40-fold in response to drought stress. For glycine betaine (Figure 8D), there was a 0.7-fold increment. After the rehydration period, proline, and glycine betaine (Figure 8A,C) increased their concentrations by 16- and 0.47-fold, respectively, compared with control plants.

The concentration of total soluble sugars (Figure 8E) also increased in plants under drought stress, regardless of the atmospheric CO_2_ concentration. After the rehydration period (Figure 8F), all plants showed values as those of the control plants.

#### 3.2.3. Biochemical Parameters

**Figure 9:**
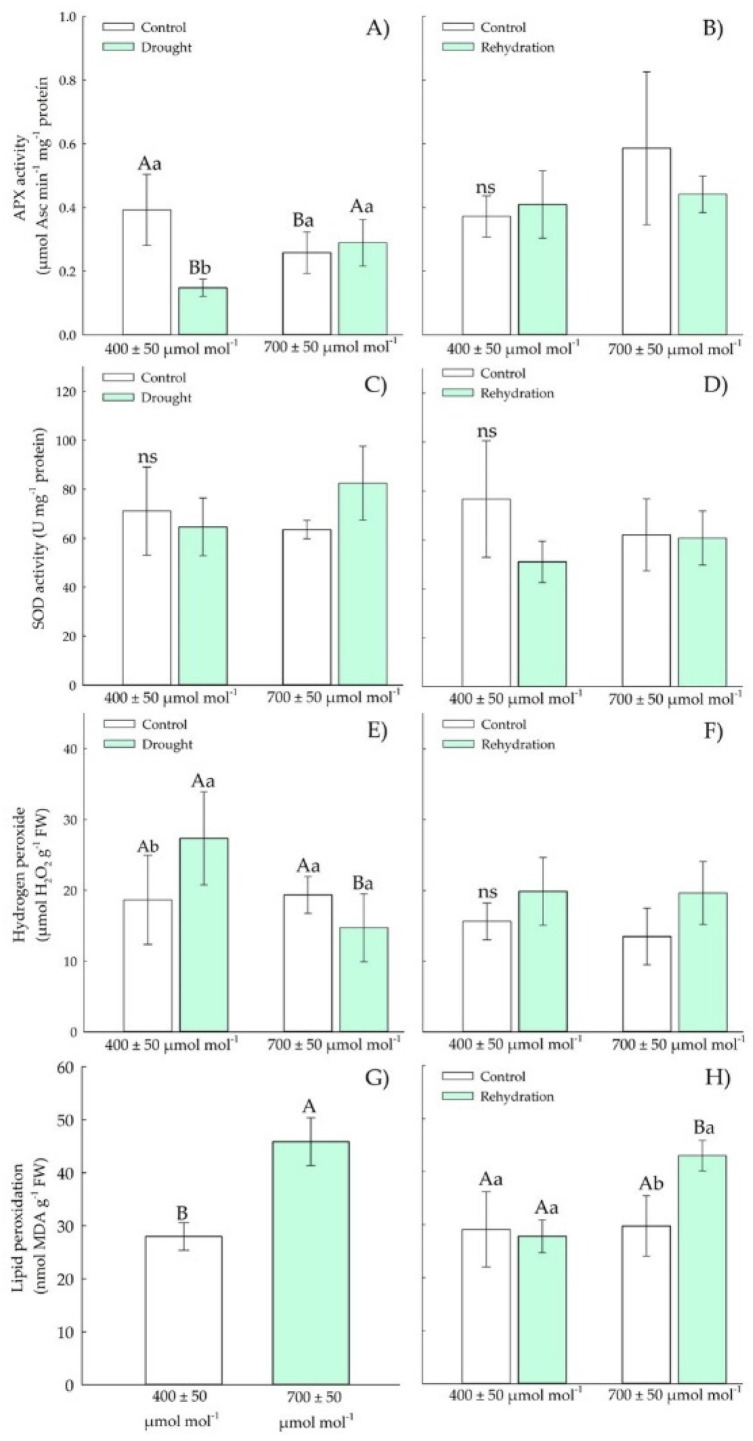
Effects of CO_2_ concentration (*a*[CO_2_] = 400 μmol mol^−1^ and *e*[CO_2_] = 700 μmol mol^−1^) and drought stress in *A. buniifolius* leaf APX activity (**A**,**B**), SOD activity (**C**,**D**), hydrogen peroxide (**E**,**F**), lipid peroxidation (**G**,**H**). In plants sampled drought stress (**A**,**C**,**E**,**G**) and rehydration (**B**,**D**,**F**,**H**) period. Different uppercase letters indicate a significant difference between values in different CO_2_ within the same water treatment, while different lowercase letters indicate significant differences between values of different water treatments within each CO_2_ level by Tukey’s test (*p* ≤ 0.05). ^ns^ denotes non-significance (*p* > 0.05). Error bars represent a 95% confidence interval.

Chirca control plants were exposed to drought stress and *a*[CO_2_] increased APX activity. After the rehydration period, APX activity (Figure 9B) was as that of control plants. Chirca plants subjected to water deficit presented SOD activity (Figure 9C) values as those of control plants. However, after rehydration, the plants had their SOD activity (Figure 9D) under that of control plants.

**Figure 10:**
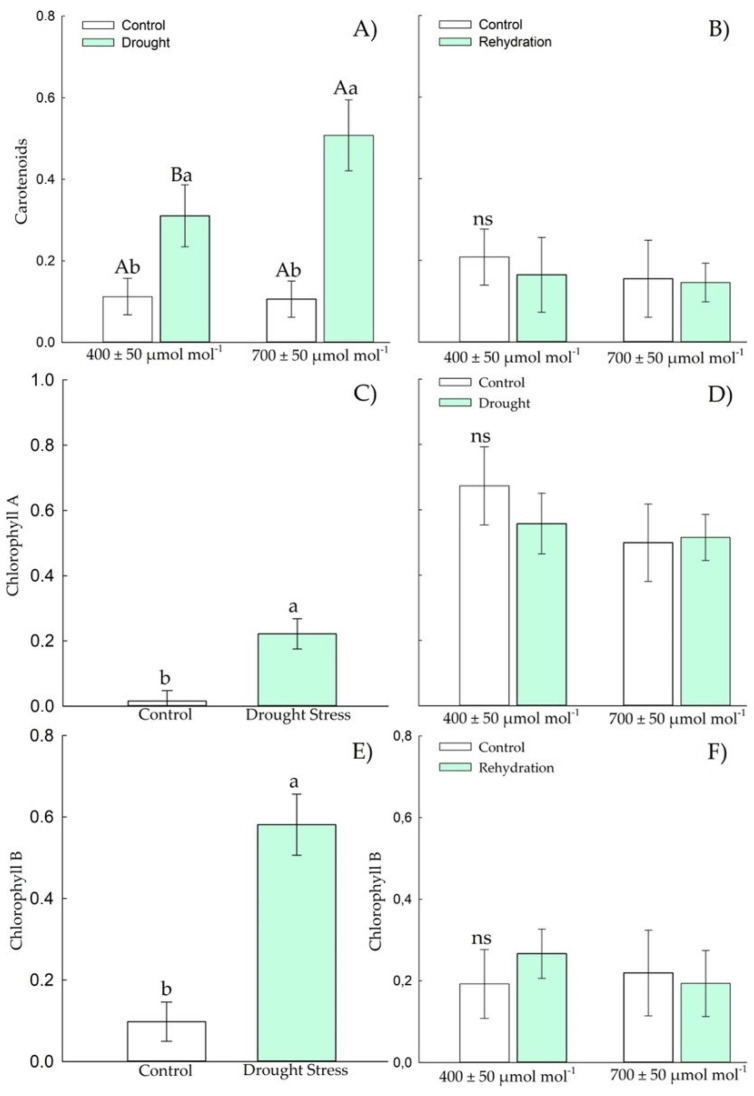
Effects of CO_2_ concentration (*a*[CO_2_] = 400 μmol mol^−1^ and *e*[CO_2_] = 700 μmol mol^−1^) and drought stress in *A. buniifolius* leaf carotenoids content (**A**,**B**), chlorophyll A (**C**,**D**), chlorophyll B (**E**,**F**). In plants sampled drought stress (**A**,**C**,**E**) and rehydration (**B**,**D**,**F**) period. Different uppercase letters indicate a significant difference between values in different CO_2_ within the same water treatment, while different lowercase letters indicate significant differences between values of different water treatments within each CO_2_ level by Tukey’s test (*p* ≤ 0.05). ^ns^ denotes non-significance (*p* > 0.05). Error bars represent a 95% confidence interval.

The concentration of hydrogen peroxide (Figure 10E) in plants exposed to drought stress and elevated CO_2_ decreased relative to drought at ambient CO_2_. During rehydration, no differences were observed between treatments (Figure 9F). Lipid peroxidation (Figure 9G) increased in Chirca plants exposed to *e*[CO_2_], regardless of drought stress. However, during the rehydration period (Figure 9H), an increase in lipid peroxidation was only observed in plants under *e*[CO_2_]. Chirca plants had an increase in carotenoids (Figure 10A) when exposed to water and *e*[CO_2_] deficit. However, after the rehydration period (Figure 10B), all plants showed values as those of the control plants.

Chlorophyll A and B (Figure 10C,E) increased in plants exposed to drought stress. After the rehydration period (Figure 10D,F), the Chirca plants showed values as those of the control plants.

#### 3.2.4. Root Dry Mass

**Figure 11:**
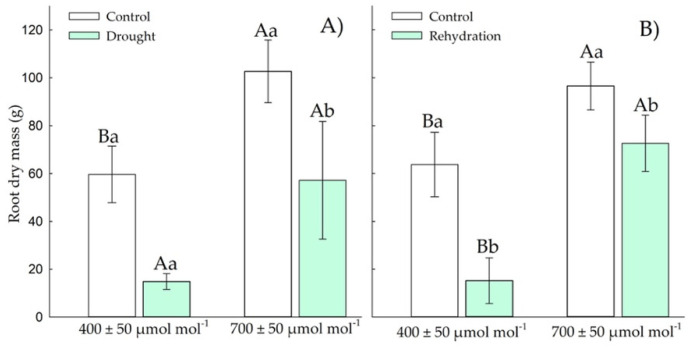
Effects of CO_2_ concentration (*a*[CO_2_] = 400 μmol mol^−1^ and *e*[CO_2_] = 700 μmol mol^−1^) and drought stress in *A. buniifolius* in root dry mass. In plants sampled after drought stress (**A**) and rehydration (**B**) period. Different uppercase letters indicate a significant difference between values in different CO_2_ within the same water treatment, while different lowercase letters indicate significant differences between values of different water treatments within each CO_2_ level by Tukey’s test (*p* ≤ 0.05). ^ns^ denotes non-significance (*p* > 0.05). Error bars represent a 95% confidence interval.

Elevated CO_2_ increased root biomass regardless of drought status. Chirca plants had an increase in root dry mass (Figure 11A,B) in *e*[CO_2_], both in the period of drought stress and rehydration.

The control plants of Chirca, when exposed to *a*[CO_2_], had an increase of 0.017 g day^−1^; the plants exposed to drought stress decreased their root dry mass by 0.020 g day^−1^ from the vegetative period to the beginning of the reproductive period. When exposed to *e*[CO_2_], control plants decreased by 0.035 g day^−1^, and plants under drought stress increased by 0.053 g day^−1^ from the vegetative period to the beginning of the reproductive period.

## 4. Discussion

Chirca is a problematic plant, which has been causing a decline in the quality of natural pastures in the Pampa Biome [7,8]. It is easily adaptable [11,37] and difficult to manage [38]. Climate change may favor this adaptation process, as found in this study where there was an increase in the root system.

In the vegetative period, Chirca plants exposed to drought stress and *e*[CO_2_] took five days longer to reach 15% of pot capacity than plants under *a*[CO_2_]. C_3_ plants benefit from the increase in [CO_2_] as the carboxylation activity of Rubisco is enhanced, increasing net photosynthesis, in addition to reducing stomatal conductance, decreasing transpiration, and reducing photorespiration [39,40]. On the other hand, the response in the reproductive period was the opposite; plants exposed to *a*[CO_2_] took one day longer to reach 15% of pot capacity than plants exposed to *e*[CO_2_]. The response to the increased concentration of CO_2_ in the atmosphere depends on the plant species, the type of photosynthetic metabolism of the plant, and environmental conditions such as water availability [41]. Weeds can also be sensitive to water deficits in the reproductive phase. In the reproductive period, plants subjected to water stress may not show plasticity for recovery [42].

The water deficit induced in this study (15% of the pot capacity) was sufficient to reduce the relative water content of Chirca plants in *e*[CO_2_]. It caused a reduction in the chemical potential of water both in the vegetative stage and in the onset of reproductive activity (Figure 3 and Figure 8). The reduction in water’s chemical potential and relative water content is a primary sign of water stress, affecting the movement and availability of water in the plant [43].

Our results indicated that the accumulation of osmolytes in Chirca under water deficit and *e*[CO_2_] (Figure 4 and Figure 9) may have represented an attempt to maintain cell turgor, preventing RWC from reaching critical levels (below 50%) and possibly causing severe and irreversible physiological damage [44]. One of the main adaptations that result in drought tolerance is the osmotic adjustment. The osmotic adjustment corresponds to the decrease in the osmotic potential, by the accumulation of compatible solutes, in response to water deficit and salinity that allows the maintenance of positive turgor at relatively low water potentials [45]. Therefore, the accumulation of osmolytes aims to reduce the cell’s water potential below the external water potential, allowing the water to move into the cell and be kept there and preventing dehydration.

Proline content is a crucial component of osmotic adjustment and an antioxidant protector in the cell (Figure 3A,B and Figure 8A,B). Proline accumulation is a physiological-biochemical indicator of drought stress, and the increase in its concentration can be correlated with high drought tolerance [46]. The synthesis of proline, when under normal conditions, occurs in the cytosol; however, when under stress conditions, it also occurs in the chloroplasts [47]. Other functions are correlated with the accumulation of this osmolyte, such as carbon and nitrogen reserve used in recovery after stress, detoxification of excess ammonia, protein and membrane stabilizers, and elimination of ROS [48,49].

In our study, plants in the vegetative stage subjected to *e*[CO_2_] and drought stress showed greater proline accumulation than the other treatments (Figure 4A). Even after rehydration (four days after stress), the proline content was still high (Figure 4B). At the beginning of the reproductive period, plants under water deficit produced higher proline accumulation than control plants (Figure 8A). The increase in proline production is a metabolic strategy, considered one of the first responses of plants to try to reduce water changes in cells [50], in addition to acting in osmotic regulation and against the harmful effects produced by reactive oxygen species. The accumulation of proline in leaves and plants under water deficit may be related to the decrease in the osmotic potential of water use efficiency [51,52].

As the water deficit intensifies, the balance between water uptake and water loss becomes more difficult for plants, and new strategies are needed for plants to be able to tolerate drought. Betaine glycine, another protective osmolyte, was accumulated in Chirca plants under *e*[CO_2_] subjected to water deficit in the vegetative period (Figure 4C) and under rehydration in the early reproductive period (Figure 8D). Glycine betaine is the best-known quaternary ammonium compound in cultivated plants, endogenously synthesized in chloroplasts in response to abiotic stresses such as water deficit [53]. Its primary function is to protect the thylakoid membranes, which maintains the photochemical efficiency in photosynthesis [54]. Glycine betaine provides osmoprotection to plants by decreasing the osmotic potential, which helps to maintain adequate water absorption and increase cell turgor, protecting cells from dehydration during water stress [54,55,56].

Our results showed that the exposure of Chirca to rehydration and *e*[CO_2_] in the vegetative period and a water deficit at the beginning of the reproductive period, as well as a greater accumulation of total soluble sugars (Figure 4D and Figure 8E), likely reinforcing the osmotic adjustment. Plants exposed to water deficit can adjust their osmolality by accumulating soluble sugars [57,58]. Carbohydrates accumulated during stress are important to plants in three ways: for use in regeneration, for making new structural components, and for osmotic adjustment and reduction of oxidative damage [59,60].

The adverse effects of water deficit on photosynthesis, protein denaturation, and cell leaking are primarily due to (and also reinforcing) the overproduction of ROS and lipid peroxidation of membranes [61]. It was observed that the increase in lipid peroxidation occurred both in the vegetative stage in water deficit and rehydration (Figure 5G,H). Changes in membranes, resulting from lipid peroxidation, lead to permeability disorders, changing the ionic flux and the flux of other substances, resulting in the loss of selectivity for the entry and exit of nutrients and toxic substances in the cells, and eventually causing cell death in more severe stress situations [62,63].

Low RWC values (Figure 2G) may have triggered the induction of the antioxidant system, confirmed by the increased activity of APX and SOD (Figure 4A,C) under water deficit in the vegetative period in Chirca. APX in the AsA-GSH enzyme cycle plays a vital role in neutralizing the production of H_2_O_2_ generated by SOD in different cell organelles [64], which could have prevented the accumulation of hydrogen peroxide in Chirca (Figure 4E). However, this response was not observed in the stage of the beginning of the reproductive cycle, where the activity of APX and SOD were similar in control plants and plants that underwent exposure to water deficit and rehydration (Figure 10). The accumulation of hydrogen peroxide was probably enough to cause lipid peroxidation in Chirca under drought stress (Figure 9G). However, the accumulation of the osmoprotectants (proline, glycine betaine, and total soluble sugars) may have contributed to stabilizing the membrane and preventing damage from the accumulation of H_2_O_2_ since the plants exhibited an excellent capacity for recovery after rehydration.

The water deficit in the vegetative stage caused an increase in H_2_O_2_ (Figure 4E); meanwhile, at the beginning of the reproductive period, there was a decrease in H_2_O_2_ in plants exposed to water deficit and *e*[CO_2_] (Figure 9E). We hypothesized that, although plants in both populations produced similar H_2_O_2_ values, plants grown in *e*[CO_2_] have a higher capacity to neutralize the deleterious effects of ROS accumulation. Enzyme protection is based almost exclusively on superoxide anion decomposition or dismutation of hydrogen peroxide, oxidizing agents. Additionally, macromolecules such as tocopherols, flavonoids, and carotenoids also play a pivotal role in preventing the adverse oxidative effects of ROS [63]. The accumulation of carotenoids (Figure 5A and Figure 10A) found in Chirca plants under drought stress, both in the vegetative and reproductive stages, indicates the role of the non-enzymatic system helping in redox homeostasis. To minimize the effects of oxidative stress, plants have evolved a complex non-enzymatic, low-molecular-mass antioxidant system such as carotenoids [61]. Carotenoids scavenge singlet oxygen (^1^O_2_) produced in thylakoid membranes by PSII acts as both visible and UV light filters, reducing cell damage caused by excessive light energy [65].

Although plants showed significant responses of the enzymatic and non-enzymatic defense system, an increase in lipid peroxidation was observed in plants subjected to drought stress, even after rehydration, especially in the vegetative stage under *e*[CO_2_], and also after rehydration in the stage of the beginning of the reproductive cycle (Figure 4G,H and Figure 9G,H). However, the oxidative damage was not severe enough to cause significant effects on the content of chlorophylls A and B in the vegetative and early reproductive stages (Figure 5C,E and Figure 10C,E). Chlorophyll biosynthesis is down-regulated by water stress; therefore, this could act as a regulatory mechanism in plants to resist drought, minimizing light absorption due to reduced amounts of chlorophyll that would negatively regulate electron transport and reduce the production of ROS [66]. However, such a possibility was not evident in our study (Figure 5C,E and Figure 10C,E).

Another important response mechanism when plants are exposed to water deficit is allocating resources for root growth, allowing the expansion of the water uptake area. Control plants of Chirca exposed to *e*[CO_2_] decreased the root growth rate by 0.035 g/day^−1^, while plants under water deficit increased by 0.053 g/day^−1^ from the vegetative period to the beginning of the reproductive period (Figure 6A,B and Figure 11A,B). When exposed to *a*[CO_2_], plants showed an increase of 0.017 g/day^−1^, while plants exposed to water deficit decreased root dry mass accumulation by 0.020 g/day^−1^ from the vegetative period to the beginning of the reproductive period.

When water becomes a limiting factor, more plant assimilates can be allocated to the underground system to continuously sustain root growth. This change in root architecture can be considered a line of defense against desiccation [67,68]. The excess of photoassimilates produced under high CO_2_, especially in plants with C3 metabolism [69], can be preferentially allocated to the roots playing a dual role, as a transient carbon and energy store, and also contributing to the osmotic adjustment in response to water deficit by increasing the water uptake [70]. Thus, C3 plants in *e*[CO_2_] may have an efficient regulation of the stomatal opening and, therefore, better efficiency in the assimilation of CO_2_. The increase in this efficiency under water deficit can support the higher allocation of photoassimilates to the root system [71].

## 5. Conclusions

Among the biochemical changes observed in the plants under drought stress, the accumulation of proline, glycine betaine, and total soluble sugars were the most evident mechanisms allowing plants to tolerate drought stress by osmotic adjustment. The increase in the atmospheric concentration of *a*[CO_2_] to *e*[CO_2_] stimulated growth and root dry mass

This result shows that climate change may favor this weed and may increase its presence and damage in the grasslands. Future research must evaluate the effect of climate change on this plant at the community level.

## Data Availability

The data presented in this study are available on request from the corresponding author.

## Supplementary Materials

The following are available online at https://www.mdpi.com/article/10.3390/plants11172270/s1, Table S1: Vegetative stage, Table S2: Reprodutive stage.
