# Supplementary material for: High Atmospheric CO2 Concentration Mitigates Drought Effects on Acanthostyles buniifolius an Important Grassland Weed in South America
Source: Plants (Basel). 2022 Aug 31;11(17):2270. doi: 10.3390/plants11172270 (PMC9459995; doi:10.3390/plants11172270)
Supplement: Supplementary file 1 [file plants-11-02270-s001.zip › plants-1871794-supplementary.pdf]

Supplementary Material

Table S1 **Vegetative stage**

Vegetative stage										
**Variable**	***e*[CO_2]**	***a*[CO_2]**	**Drought stress**	**Control**	***e*[CO_2] + drought stress**	***a*[CO_2] + drought stress**	***e*[CO_2] + rehydration**	***a*[CO_2] + rehydration**	**Control**	**Rehydration**
**Water potential pre daw**					↑	↓			≈	≈
**Water potential midday**			↑	↓					≈	≈
**Relative water content**			↓	↑					≈	≈
**Proline**					↑	↓	↑	↓		
**Glycine betaine**					↑	↓	≈	≈		
**Soluble sugar**					≈	≈	↑	↓		
**APX activity**	↑	↓					↑	↓		
**SOD activity**	↑	↓					↑	↓		
**Hydrogen peroxide**			↑	↓					≈	≈
**Lipid peroxidation**			↑	↓					↑	↓
**Carotenoids**					↓	↑			≈	≈
**Chlorophyll A**			↑	↓					≈	≈
**Chlorophyll B**							≈	≈		
**Root dry mass**					↑	↓			≈	≈

Qualitative table to demonstrate how the trends of the results expressed in the figures. The arrows indicate ↑ (increase), ↓ (decrease) and ≈ (similarity) when compared: ***e*[CO_2] X *a*[CO_2]; Drought stress X Control; *e*[CO_2] + drought stress X *a*[CO_2] + drought stress; *e*[CO_2] + rehydration X *a*[CO_2] + rehydration e Control X Rehydration**

Table S2 **Reprodutive stage**

**Reprodutive stage**										
**Variable**	***e*[CO_2]**	***a*[CO_2]**	**Drought stress**	**Control**	***e*[CO_2] + drought stress**	***a*[CO_2] + drought stress**	***e*[CO_2] + rehydration**	***a*[CO_2] + rehydration**	**Control**	**Rehydration**
**Water potential pre daw**			↑	↓					↓	↑
**Water potential midday**			↑	↓					↓	↑
**Relative water content**					↑	↓			↑	↓
**Proline**			↑	↓					↓	↑
**Glycine betaine**			↑	↓					↓	↑
**Soluble sugar**			↑	↓			≈	≈		
**APX activity**					↓	↑	≈	≈		
**SOD activity**					≈	≈	≈	≈		
**Hydrogen peroxide**					↓	↑	≈	≈		
**Lipid peroxidation**	↑	↓					↑	↓		
**Carotenoids**					↑	↓	≈	≈		
**Chlorophyll A**			↑	↓			≈	≈		
**Chlorophyll B**			↑	↓			≈	≈		
**Root dry mass**					↑	↓	↑	↓		

Qualitative table to demonstrate how the trends of the results expressed in the figures. The arrows indicate ↑ (increase), ↓ (decrease) and ≈ (similarity) when compared: ***e*[CO_2] X *a*[CO_2]; Drought stress X Control; *e*[CO_2] + drought stress X *a*[CO_2] + drought stress; *e*[CO_2] + rehydration X *a*[CO_2] + rehydration e Control X Rehydration**
